# Exiguolysin, a Novel Thermolysin (M4) Peptidase from *Exiguobacterium oxidotolerans*

**DOI:** 10.3390/microorganisms12112311

**Published:** 2024-11-14

**Authors:** Brendan F. Gilmore, Tracy A. White, Alessandro Busetti, Matthew I. McAteer, Christine A. Maggs, Thomas P. Thompson

**Affiliations:** 1 Biofilm Research Group, School of Pharmacy, Queen’s University Belfast, Medical Biology Centre, 97 Lisburn Road, Belfast BT9 7BL, UK; 2 School of Medicine, University of Limerick, Limerick V94 T9PX, Ireland; 3 Institute for Global Food Security, School of Biological Sciences, Queen’s University Belfast, 19 Chlorine Gardens, Belfast BT9 5DL, UK

**Keywords:** *Exiguobacterium oxidotolerans*, metalloprotease, thermolysin, protease-activated receptors

## Abstract

This study details a comprehensive biochemical and structural characterization of exiguolysin, a novel thermolysin-like, caseinolytic peptidase secreted by a marine isolate of *Exiguobacterium oxidotolerans* strain BW26. Exiguolysin demonstrated optimal proteolytic activity at 37 °C and pH 3, retaining 85% activity at 50 °C, highlighting its potential stability under broad reaction conditions. SDS-PAGE and LC-MS analysis identified the enzyme as a 32 kDa M4-family metalloprotease. Exiguolysin activity was inhibited by 1,10-phenanthroline, confirming its dependence on metal ions for activity. Zymographic analysis and substrate specificity assays revealed selective hydrolysis of matrix metalloproteinase (MMP) substrates but no activity against elastase substrates. Analysis of the predicted gene sequence and structural predictions using AlphaFold identified the presence and position of HEXXH and Glu-Xaa-Xaa-Xaa-Asp motifs, crucial for zinc binding and catalytic activity, characteristic of ‘Glu-zincins’ and members of the M4 peptidase family. High-throughput screening of a 20 × 20 *N*-alpha mercaptoamide dipeptide inhibitor library against exiguolysin identified SH-CH_2_-CO-Met-Tyr-NH_2_ as the most potent inhibitor, with a *K*i of 1.95 μM. Notably, exiguolysin selectively inhibited thrombin-induced PAR-1 activation in PC-3 cells, potentially indicating a potential mechanism of virulence in modulating PAR-1 signalling during infection by disarming PARs. This is the first detailed characterization of a peptidase of the M4 (thermolysin) family in the genus *Exiguobacterium* which may have industrial application potential and relevance as a putative virulence factor.

## 1. Introduction

The genus *Exiguobacterium* was first proposed by Collins et al. (1983), with the reclassification of a previously isolated Gram-positive, alkalophilic bacterium, *E. aurantiacum* [1]. *E. aurantiacum* was isolated from alkaline waste streams in a potato processing plant and exhibited growth in culture between 7 °C and 43 °C (optimal growth at 38 °C) and a pH growth range between pH 8–10.5. To date, the genus *Exiguobacterium* comprises over 20 diverse species, with many isolated from extreme environments, leading to their broader classification as extremophilic bacteria [2]. Bacteria of this genus are coryneform, low-G + C, Gram-positive, motile, catalase-positive, facultatively anaerobic, non-spore-forming bacilli, though variable/irregular morphologies are observed in culture [3,4].

The genus *Exiguobacterium* are broadly classed as extremophilic bacteria, with species isolated from environments as diverse as soil, marine environments [5], sediments [6], ancient permafrost [7], glaciers [8], heavily (hydrocarbon, pharmaceutical) contaminated environments [9], and hydrothermal vents [10]. These bacteria, and extremophiles generally, are of significant industrial interest due to their metabolic versatility, which includes the production of enzymes capable of functioning in harsh conditions [2,11,12]. This characteristic has led to growing interest in their use in industrial applications, particularly in enzyme production for biocatalysis under extreme conditions, in pharmaceuticals, biofuel production, and polymer degradation [3].

The demand for industrially relevant enzymes has increased significantly, with current projections estimating that the global market will reach USD 14.5 billion by 2027 [13]. Previously, a polystyrene-degrading strain, *Exiguobacterium* YT2, isolated from the gut microbiota of mealworms, was essential for mealworm survival on polystyrene diets [14]. Research on similar enzymes, such as the thermolysin-like protease A69, has shown utilised in producing bioactive peptides with antioxidative and moisture-retention properties, further illustrating the industrial appeal of such enzymes [15]. Extremozymes, such as proteases, amylases, lipases, and pullulanases, produced by the *Exiguobacterium* genus, have shown significant potential as biocatalysts in industrial applications, ranging from biopolymer breakdown to biofuel production [3]. Recently, a number of solvent-tolerant alkaline proteases of various mechanistic classes produced by diverse *Exiguobacterium* species have been predicted or isolated, which exhibit detergent activity [16], high solvent tolerance [17], halotolerance [18], and low-temperature activity [19]. However, enzymes from the *Exiguobacterium* family remain to be fully characterized in the literature. In general, bacterial thermolysin-like peptidases have been used in industrial processes but are also implicated in bacterial virulence [20]; therefore, the discovery of novel thermolysin M4 peptidases has potential for both industrial and clinical impact.

While primarily recognized for their environmental adaptability, *Exiguobacterium* species have also emerged as opportunistic pathogens, with cases of bacteraemia, wound infections, and other clinical conditions reported in immunocompromised patients [4]. The possible presence (based on 16S rRNA sequence homology) of *E. aurantiacum* in patients with periodontitis was reported in 2003, where in the microbiome of periodontal pockets of 23 patients suffering from periodontitis, 13 patients were positive for *E. aurantiacum*, indicating a potential role in periodontitis [21]. However, the first clinical case report involving this genus was the identification of *Exiguobacterium acetylicum* as the aetiological agent in a case of catheter-related bacteraemia in an elderly patient, resolved by device removal and treatment with cefuroxime [22]. Subsequently, the isolation of *Exiguobacterium aurantiacum* from blood cultures of six patients, all with varying degrees of compromised immune function, has been reported [23]. *E. aurantiacum* has also been isolated from a patient with bacteraemia following radical prostatectomy [24]. More recently, a case of cutaneous infection caused by *Exiguobacterium sibericum*, potentially of zoonotic origin, was reported in a patient presenting with ulceration with painful eschar [25]. The species *Exiguobacterium* sp. A1b/GX59 was isolated from a patient suffering severe community-acquired pneumonia and bacteraemia [26]. Importantly, this study used genomic analysis to identify a number of genes involved in pathogenicity and virulence, including genes associated with secretion systems for toxin export, uptake of DNA for acquisition of virulence and resistance, haemolysis, quorum sensing, motility, biofilm formation, and adenine methylation [26]. More recently, *Exiguobacterium profundum* was isolated as a component of the hand-sanitizer-tolerant microbiome of volunteers (following application of hand sanitizer), and exhibited antibiotic resistance to >5 antibiotics, underscoring their capacity to survive harsh environmental conditions and potentially act as a reservoir for antibiotic resistance genes [27].

The alkaliphilic species *E. oxidotolerans*, first described in 2004, was isolated from a fish processing plant drain [28] and was later detected in the oral cavities of cancer patients but found to be absent in healthy individuals [29]. More recently, studies comparing the intratumoral microbiomes of pancreatic ductal adenocarcinoma with adjacent non-cancerous tissues revealed a higher abundance of *Exiguobacterium* in tumour regions. Notably, a lower *Exiguobacterium/Bacillus* ratio correlated with better patient prognosis [30]. These findings suggest that the *Exiguobacterium* genus, while understudied, could play a significant role in the tumour microbiome, potentially influencing disease outcomes. Additionally, the genus represents a growing group of opportunistic pathogens, emphasizing the need for further research into its virulence factors and pathogenic mechanisms.

In this study, we report the isolation and characterization of a thermolysin-like metallopeptidase, Exiguolysin, from a strain of *E. oxidotolerans* associated with the marine brown seaweed *Pelvetia canaliculata* (channelled wrack) from the intertidal zone of the Irish Sea, Northern Ireland. The discovery of Exiguolysin expands our understanding of the diversity of secreted enzymes, specifically peptidases, in this genus and introduces a novel member of the M4 family of metallopeptidases, with potential relevance to both industrial and clinical scenarios. Thermolysin (M4) peptidases are widely distributed in bacteria, where they are important mediators of diverse physiological processes, and act as virulence factors implicated in infections of significant clinical concern [31]. Thermolysin toxins are involved in numerous infections and associated pathologies, including Legionnaire’s disease, peptic ulcer, gastritis, gastric carcinoma, activation of clostridial λ-toxin, degradation of host immunoglobulins, pathogenesis of cholera, biofilm formation and virulence in *P. aeruginosa*, and *S. aureus* and *E. faecalis* infection progression [20,31,32,33,34]. The discovery and comprehensive biochemical, molecular, and genomic characterization of a novel M4-family peptidase in the genus *Exiguobacterium*, increasingly implicated in infectious disease, broadens our knowledge of thermolysin-like peptidases as potential virulence factors.

The enzyme was purified and determined to be a 32 kDa M4-family peptidase. In vitro, exiguolysin efficiently hydrolysed azocasein and a synthetic fluorogenic matrix metalloproteinase (MMP) substrate, and was effectively inhibited by 1,10-phenanthroline and *N*-alpha mercaptoamide dipeptide inhibitors previously screened against the *P. aeruginosa* M4-family peptidase elastase, LasB [32,33], and mirabilysin/ZapA from *Proteus mirabilis* [35,36], confirming the classification of this enzyme as a metallopeptidase. Its ability to disarm PAR-1 may indicate a potential role in *Exiguobacterium* virulence.

The identification and characterization of a novel, thermolysin-like peptidase from *E. oxidotolerans* demonstrates the first characterization of a novel secreted M4-family peptidase in this genus, and whilst the precise physiological function of the enzyme remains to be elucidated, members of the M4 peptidase family have been implicated as virulence factors in a wide range of bacterial infections [31]. Therefore, we propose exiguolysin as a potential virulence factor from *E. oxidotolerans*.

## 2. Materials and Methods

### 2.1. Sample Collection and Bacterial Isolation

A sample of the common brown seaweed *Pelvetia canaliculata* from the intertidal zone of the Irish Sea, Bangor West, was collected from the east coast of Northern Ireland. To dislodge surface-associated bacteria, samples were suspended in 10 mL of sterile phosphate-buffered saline (PBS) and vortexed vigorously. Serial dilutions were prepared, and 100 µL of each dilution was spread onto nutrient agar supplemented with 2.5% NaCl, nutrient agar (10%), and lysogeny broth (LB) agar containing 2% skim milk for caseinolytic activity screening. Plates were incubated at 5 °C, 15 °C, and 27 °C in triplicate to assess growth across different temperature ranges. Skim milk was used to quickly identify caseinolytic peptidase-secreting isolates.

### 2.2. Azocasein Hydrolysis Assay

Peptidase activity was quantified using azocasein hydrolysis. Conditioned media (100 µL) was mixed with 1% (*w*/*v*) azocasein in PBS and incubated at 27 °C for 1 h. The reaction was quenched by adding 400 µL of 5% trifluoroacetic acid (TFA), and the mixture was centrifuged at 13,000 rpm for 10 min. Absorbance of the supernatant was measured at 440 nm to determine protease activity. Units of protease activity were calculated as previously described by Loomes et al. [37].

### 2.3. Growth Conditions, and 16S rRNA Identification

Isolates were grown in LB at 27 °C with agitation (100 rpm) for 48 h. DNA was extracted using the GeneElute^®^ Bacterial Genomic DNA Kit (Sigma Aldrich, St. Louis, MO, USA) following the manufacturer’s protocol. The 16S rRNA gene was amplified using standard primers 27F (5′-AGA GTT TGA TCM TGG CTC AG-3′) and 1492R (5′-TAC GGY TAC CTT GTT ACG ACT T-3′). The gene was sequenced via Sanger sequencing perfomed by Eurofins Genomics (Wolverhampton, UK) in both directions, providing robust coverage of the central region of the gene, while the 5′ and 3′ ends were covered by single-strand sequencing. Sequences were aligned against the NCBI GenBank database using BLAST (Basic Local Alignment Search Tool) to confirm isolate identification.

### 2.4. Phylogenetic Analysis

16S rRNA sequences were aligned using the MUSCLE algorithm in MEGA11 [38]. A phylogenetic tree was constructed using the Maximum Likelihood method with the Tamura–Nei model. Bootstrap analysis (100 replicates) was used to assess the confidence of branch points. Sequences with significant similarity to the *Exiguobacterium* genus were included in the analysis

### 2.5. Protease Characterization and Caseinolytic Activity

Overnight cultures were centrifuged (4000 rpm, 4 °C, 12 min), and the supernatant was filtered through a 0.45 µm nitrocellulose membrane. Protease activity was evaluated by incubating 100 µL of supernatant with 1% azocasein at 37 °C for 1 h. The reaction was quenched with 400 µL of TFA, and absorbance was measured at 440 nm. Protease mechanistic class was determined by incubating the supernatant with 2 mM 1,10-phenanthroline (metalloprotease inhibitor), 0.2 mM PMSF (serine protease inhibitor), and 10 µM E-64 (cysteine protease inhibitor) prior to azocasein hydrolysis. 

### 2.6. Protease Purification by Hydrophobic Interaction Chromatography (HIC)

Proteins from the bacterial supernatant were precipitated by adding ammonium sulphate to a final concentration of 500 mM at 4 °C. Precipitated proteins were applied to a HiTrap^®^ Phenyl Sepharose HP column (GE Healthcare, Chalfont St. Giles, UK) pre-equilibrated with 50 mM Tris buffer containing 1 M ammonium sulphate and 20% glycerol (pH 7.5) using an Akta Prime system. Unbound proteins were removed by washing with equilibration buffer until a stable baseline was reached. Proteins were eluted with a linear gradient of Tris buffer (pH 7.5) from 50% to 100% in 10% increments. Eluted fractions were assayed for proteolytic activity using the fluorogenic substrate Mca-Lys-Pro-Leu-Gly-Leu-Dap-(Dnp)-Ala-Arg-NH2. Active fractions were concentrated by ammonium sulphate precipitation, followed by dialysis against 50 mM Tris buffer (pH 7.5). Proteins were re-applied to the same HIC column under identical conditions to improve purity. Fractions were collected in 1 mL aliquots, immediately chilled on ice, and stored at −20 °C. Proteolytic activity of individual fractions was confirmed by substrate hydrolysis assays. SDS-PAGE was used to assess the purity of protease fractions. Samples were mixed with Laemmli buffer, boiled at 95 °C for 5 min, and loaded onto a 12% polyacrylamide gel. Electrophoresis was carried out at 100 V for 1.5 h. Gels were stained with Coomassie Blue and destained in a methanol–acetic acid solution.

### 2.7. Temperature and pH Profiling

Peptidase stability of the crude extract was evaluated by conducting azocasein assays at temperatures ranging from −20 °C to 50 °C. For pH profiling, enzyme reactions were performed in buffers ranging from pH 3 to pH 9, using sodium acetate (pH 3–5), phosphate (pH 6–7), and Tris-HCl (pH 8–9) buffers. Relative protease activity was calculated by comparison to activity at the optimal temperature (27 °C) and pH (7.5).

### 2.8. Extraction of Exiguolysin from Solid Growth Media

Petri dishes containing LB agar supplemented with 2% skim milk were streaked with *E. oxidotolerans* and grown at 27 °C for 48 h. The zones of proteolysis were identified by the clear halos surrounding bacterial growth. To extract the protease, the clear zones were excised, diced (~3.5 g), and suspended in 10 mL of PBS. Control plates with no zones of clearance were processed similarly. The agar/PBS mix was agitated overnight at 4 °C at 180 rpm, and the agar was removed by centrifugation at 4000 rpm for 10 min at 4 °C. The supernatant was filtered using a Whatman Grade 1 glass fibre filter to remove residual agar pieces. The protein concentration was measured using the Qubit™ Protein Quantification System (Invitrogen, Paisley, UK). The filtrate was concentrated using Amicon Ultra centrifugal filters and then analyzed by SDS-PAGE and LC-MS/MS at the Fingerprint Proteomics Facility, University of Dundee, Scotland, UK.

A modified extraction method involved growing an overnight culture of *E. oxidotolerans* in LB at 27 °C (100 rpm), filtering through a 0.2 µm nitrocellulose membrane, and placing the inoculated membrane on an LB agar plate with 2% skim milk. After 18 h at room temperature (23–25 °C), the clear zones beneath the membrane were excised and processed for protease extraction as described.

### 2.9. Bio-Assay-Guided Fractionation and Activity Verification

Proteolytic activity was determined by hydrolysis of the fluorogenic matrix metalloprotease (MMP) substrate Mca-Lys-Pro-Leu-Gly-Leu-Dap-(Dnp)-Ala-Arg-NH2. Fractions exhibiting time-dependent substrate hydrolysis were lyophilized and stored at −20 °C. The active fractions were analyzed for purity by SDS-PAGE, and protein bands were visualized using Coomassie Blue staining. The most prominent bands were excised and subjected to LC-MS/MS analysis (using ABSciex 4000 QTRAP linear ion trap mass spectrometers with a Dionex U3000 nanoLC system) for precise identification at the Fingerprint Proteomics Facility, Dundee University, Dundee, UK. Protein identification was conducted using MASCOT database searching software.

### 2.10. Assessment of Peptidase Specificity

Peptidase activity was confirmed using the matrix metalloproteinase (MMP) substrate Mca-Lys-Pro-Leu-Gly-Leu-Dap-(Dnp)-Ala-Arg-NH2 (Peptides International). Class-specific inhibitors were used to determine the catalytic mechanism, including E-64 (10 μM) for cysteine proteases, 1,10 phenanthroline (2 mM) for metalloproteases, and PMSF (0.2 mM) for serine proteases. These concentrations were selected based on established guidelines for effective inhibition [39]. Fluorescence was monitored at excitation 330 nm and emission 460 nm over a 45 min period as described previously [33].

Time-dependent hydrolysis of the fluorogenic substrate Mca-Lys-Pro-Leu-Gly-Leu-Dap-(Dnp)-Ala-Arg-NH2 by exiguolysin was measured in 96-well plates (total volume 100 µL) with fluorescence was measured every 30 s for 45 min using a plate reader (excitation at 330 nm and emission at 460 nm). Progress curves were generated, and kinetic parameters were determined using non-linear regression.

### 2.11. Zymographic Analysis of Protease Activity

Casein zymography was used to visualize protease activity in fractions collected from the Akta Prime HIC system. Samples were prepared in Laemmli buffer and run on a Tris-Glycine casein zymogram gel using the XCell *SureLock*™ Mini-Cell system. Following electrophoresis, gels were sectioned and renatured in Novex zyogram renaturing buffer with and without 1,10 phenanthroline (5 mM) to assess metalloprotease activity. Gels were stained, destained, and analyzed for caseinolytic peptidase activity.

### 2.12. BLASTn and Sequence Similarity Analysis

The thermolysin gene sequence obtained from *E. oxidotolerans* was analyzed using the BLASTn web tool (NCBI) to assess similarity against other *Exiguobacterium* strains. BLAST searches were conducted against the NCBI GenBank database, and percentage identity was calculated for each strain. A cutoff of 70% identity was used to determine significant matches. The sequences with the highest similarity were compared, and results were tabulated to show the percentage identity for each strain.

### 2.13. Inhibitor Screening and Ki Value Determination

The inhibition of protease activity was screened using a 20 × 20 library of N-alpha dipeptide mercaptoamide inhibitors as previously described by Cathcart et al. [22], and the half-maximal inhibitory constant (*K*_i_) values were determined [32]. In order to determine Michaelis–Menten constant, *K*_m_, of the fluorogenic substrate, exiguolysin (previously purified by HIC, final concentration 5 units/well) was added to wells of a 96-well black microtitre plate (Nunc); the volume was adjusted to 75 µL in assay buffer (25 mM Tris, 2.5 mM CaCl_2_, pH 7.0), and fluorogenic substrate (25 µL) was added at concentrations of 0, 1, 10, 25, 50, 100 and 200 µM. Time-dependent hydrolysis of the fluorogenic substrate was measured (excitation/emission 300 nm/460 nm) every 30 s for 30 min. The *K*_m_ is determined using the Michealis–Menten equation, by plotting the rate of substrate hydrolysis against substrate concentration. A *K*_m_ value of 14.20 µM was subsequently used to calculate *K*_i_ values for each inhibitor.

Initially, each inhibitor was tested at a final concentration of 100 µM in a broad-spectrum screen, employing a standard fluorogenic substrate assay using Mca-Lys-Pro-Leu-Gly-Leu-Dap-(Dnp)-Ala-Arg-NH_2_. Enzyme reactions were carried out in a 96-well black plate at 37 °C, and fluorescence was monitored as described above. The resulting data were used to determine fractional inhibition, F_i_, by dividing the rate of substrate hydrolysis (Vi) in the presence of each inhibitor by the rate of hydrolysis of the positive control (V_0_) containing no inhibitor, to identify the most potent inhibitors of the M4-family thermolysin-like peptidase of *E. oxidotolerans* for comprehensive kinetic evaluation. The top ten most potent inhibitors (by fractional inhibition) for exiguolysin were selected for further characterization over an extended concentration range to determine accurate inhibition constants (*K*_i_), calculated using the Morrison equation for tight-binding inhibitors, as described previously by Carson et al. [33], on GraphPad Prism 10 (2).

### 2.14. Calcium Mobilization Assay

Calcium mobilization in PC-3 and HCT15 cells was assessed using the fluorescent calcium indicator Fluo-4 AM (ThermoFisher, UK). Cells were cultured in RPMI-1640 medium with 10% fetal bovine serum and antibiotics, maintained at 37 °C with 5% CO_2_. Cells were seeded in 96-well plates and incubated with 5 μM Fluo-4 AM for 30 min at 37 °C. After washing, cells were treated with 5 units/well of thermolysin-like protease or the agonists TFLLRN (PAR-1) [40] and SLIGKV (PAR-2) [41]. Intracellular calcium changes were monitored using a BMG Fluostar plate reader (excitation: 494 nm, emission: 516 nm).

### 2.15. AlphaFold v3 Protein Structure Prediction and Visualization

The three-dimensional structure of the thermolysin-like protease from *E. oxidotolerans* was predicted using AlphaFold v3 with default settings. The protein sequence, identified through LC-MS/MS analysis, was submitted to the AlphaFold v3 model, which leverages advanced deep learning techniques to predict protein structures from sequence data with high accuracy. The model with the highest confidence score, based on the predicted local distance difference test, was selected for further structural analysis. Key residues, such as the HEXXH zinc-binding motif and the Glu-Xaa-Xaa-Xaa-Asp motif, were annotated according to functional predictions and known structural motifs in related thermolysin-like proteases. The predicted structure was visualized using ChimeraX v1.4 to illustrate the spatial arrangement of key functional residues and the overall fold of the protein.

### 2.16. Statistical Analysis

**Figure 1:**
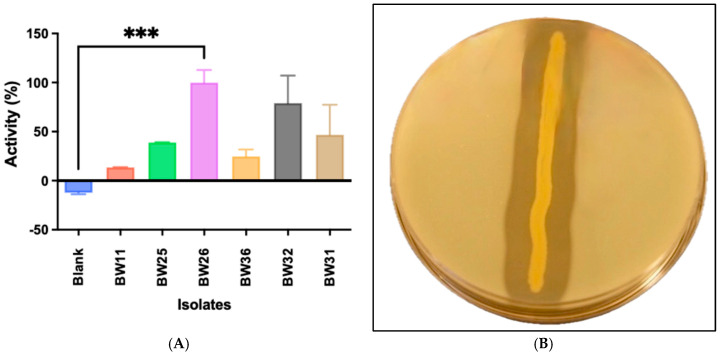

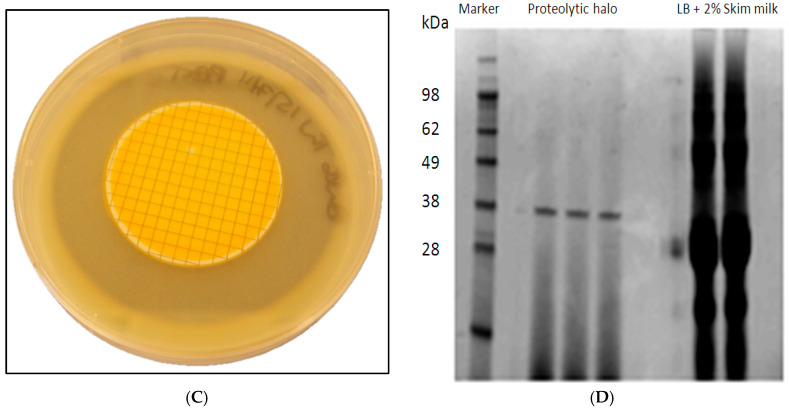
(**A**) Azocasein hydrolysis assay showing relative proteolytic activity of conditioned media from each isolate. BW26 exhibited the highest activity. Error bars represent mean ± SD from three biological replicates. Statistical significance between blank control and isolates is indicated (*** *p* < 0.001). (**B**) Proteolysis zone produced by *E. oxidotolerans* grown on LB agar with 2% skim milk at 27 °C for 48 h. (**C**) *E. oxidotolerans* grown on a nitrocellulose membrane placed on LB agar with 2% skim milk, showing proteolytic activity as a clear halo under/around the membrane. (**D**) SDS-PAGE analysis of *E. oxidotolerans* protease extracted from LB + skim milk agar. Lanes 3, 4, and 5 show the protease band at ~35 kDa. Lanes 7 and 8 serve as negative controls with LB + skim milk agar.

The results for Figures 1A, 3, and 5 we determined using a one-way ANOVA, with Dunnett’s test against the untreated control. *p* values of * (*p* ≤ 0.05), ** (*p* ≤ 0.01), *** (*p* ≤ 0.001), and **** (*p* ≤ 0.0001) indicate statistical significance. GraphPad Prism 10.3.1 was used for all analyses.

## 3. Results

### 3.1. Isolation and Screening of Protease-Producing Bacteria

Marine samples collected from the east coast of Northern Ireland were screened for peptidase (caseinolytic activity)-producing bacteria. After incubation at 5 °C, 15 °C, and 27 °C, several bacterial isolates exhibited growth across different temperatures. The proteolytic activity of conditioned media from various isolates was assessed using the azocasein hydrolysis assay. Isolate BW26 exhibited the highest level of azocasein hydrolysis compared to other isolates and the blank control (Figure 1A) and was selected for further characterisation. Statistical analysis confirmed that the activity of BW26 was significantly higher (*p* < 0.0001).

The ability of BW26 to hydrolyse casein was further demonstrated on LB agar plates supplemented with 2% skim milk, and clear zones of proteolysis were observed around the bacterial colonies after incubation at 27 °C for 48 h (Figure 1B). Additionally, when BW26 was grown on a nitrocellulose membrane placed on skim milk agar, a clear halo was visible beneath and around the membrane, indicating extracellular protease secretion (Figure 1C) and permitting removal of the bacterial colony from the agar surface to facilitate agar extraction. Proteins extracted from the proteolytic zones were analysed by SDS-PAGE. A prominent band at approximately 35 kDa was observed in lanes containing BW26 extracts (Figure 1D, lanes 3, 4, and 5), which was absent in the control lanes (lanes 7 and 8). This suggests the presence of a secreted protease corresponding to that molecular weight.

### 3.2. Identification of Exiguobacterium oxidotolerans BW26

**Figure 2:**
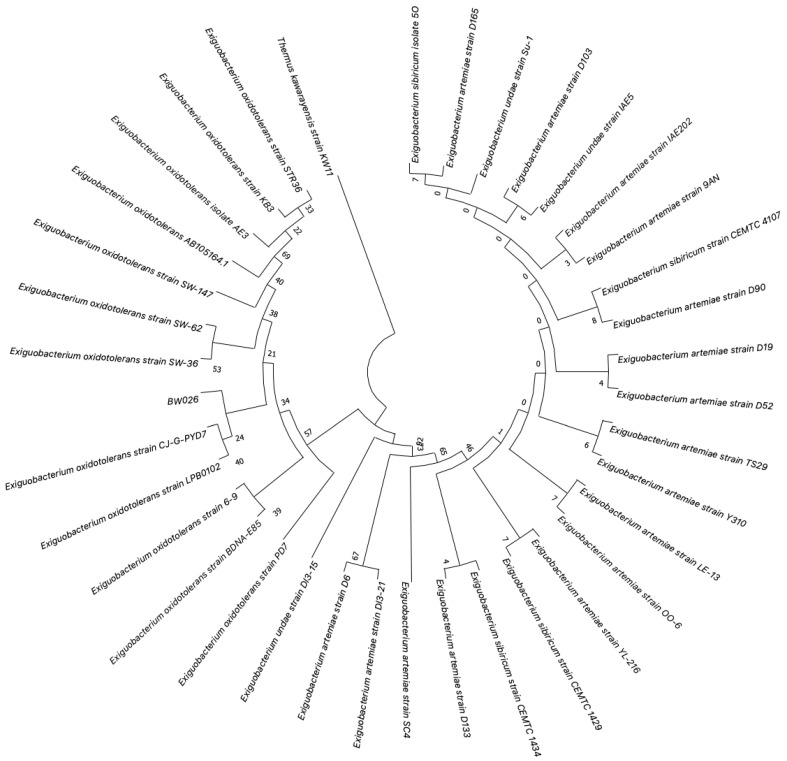
Phylogenetic tree of *Exiguobacterium* species based on 16S rRNA gene sequences. The tree was constructed using the Maximum Likelihood method with the Tamura–Nei model. Bootstrap values (1000 replicates) indicate branch reliability.

Genomic DNA extracted from isolate BW26 was used for 16S rRNA gene amplification and sequencing. BLAST analysis of the 16S rRNA sequence revealed 99.7% identity with *E. oxidotolerans* strain CJ-G-PYD7. While public databases like GenBank are commonly used for sequence alignment, we acknowledge that occasional inaccuracies in database entries may exist. The central region of the 16S gene was sequenced on both strands, while the 5′ and 3′ ends were covered by single-strand sequencing. However, the high sequence identity obtained from BLAST and the additional confirmation by proteomic validation (LC-MS/MS) reinforces the accuracy of the 16S rRNA sequence and the identification of the isolate as *E. oxidotolerans* (Figure 2). Phylogenetic analysis positioned BW26 within the *Exiguobacterium* genus, closely related to other *Exiguobacterium* species (Figure 2). This analysis provided a genetic basis for classifying the isolate within the *Exiguobacterium* genus and supported the observation of conservation of protease activity across related species.

### 3.3. Inhibition Studies and Specificity of the Protease

**Figure 3:**
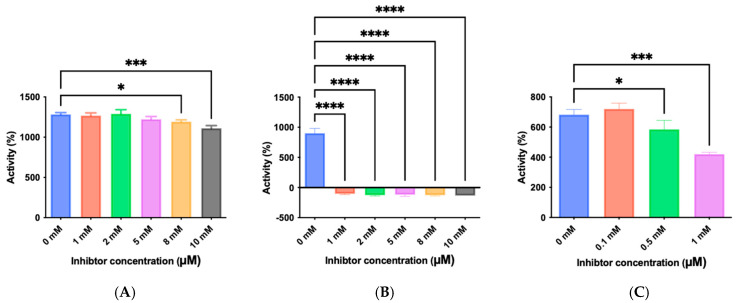
Inhibition of azocasein hydrolysis by *E. oxidotolerans* protease in the presence of class-specific inhibitors: (**A**) E-64 (10 μM), (**B**) 1,10-phenanthroline (2 mM), and (**C**) PMSF (0.2 mM). Assays were performed at 37 °C for 1 h. Error bars indicate the mean ± standard deviation determined from biological replicates; asterisks indicate significant differences between control (0 mM) and treated samples, * (*p* < 0.05), *** ( *p* < 0.001), and **** (*p* < 0.0001) using one-way ANOVA and Dunnett’s post-test analysis (*n* = *3*).

**Figure 4:**
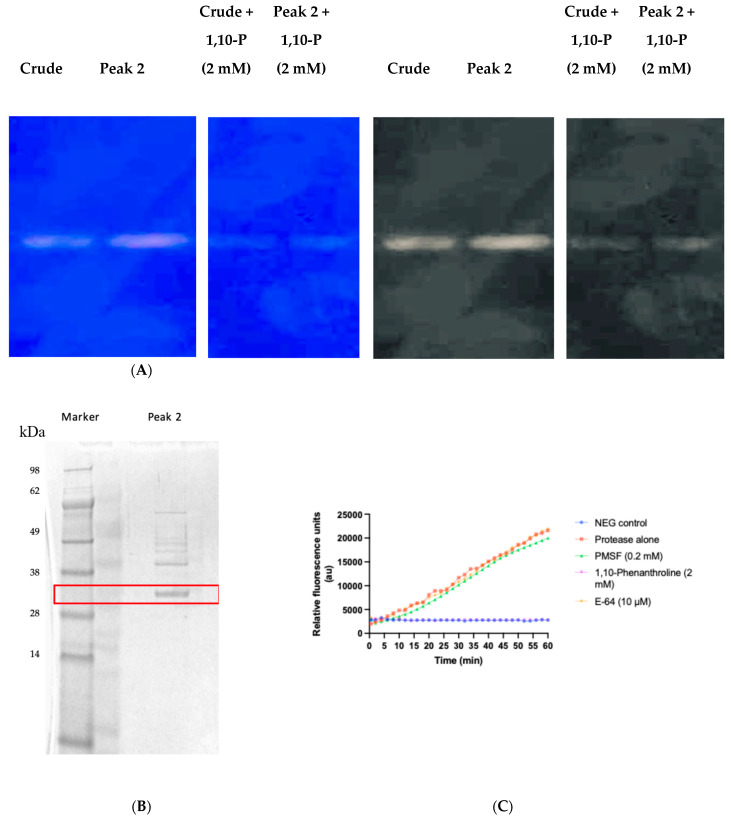
(**A**) Zymogram showing proteolytic activity of crude and partially purified fractions of *E. oxidotolerans* peptidase with and without 1,10-P (1,10-phenanthroline) (2 mM). (**B**) SDS-PAGE of chromatographic fractions with peak 2 of partially purified *E. oxidotolerans* peptidase extract, showing a band at approximately 32 kDa. (**C**) Progress curves for the time-dependant hydrolysis of the fluorogenic substrate Mca-Lys-Pro-Leu-Gly-Leu-Dap-(Dnp)-Ala-Arg-NH2 by *E. oxidotolerans* peptidase in the presence of class-specific inhibitors.

To initially determine the catalytic mechanistic class of the caesinloytic peptidase secreted by *E. oxidotolerans* BW26, its sensitivity to class-specific protease inhibitors was determined using the azocasein assay. The metalloprotease inhibitor 1,10-phenanthroline significantly reduced protease activity (*p* < 0.0001), indicating that the enzyme is likely a metalloprotease (Figure 3). Slight inhibition by PMSF (0.2 mM) and partial inhibition by E-64 (10 μM) indicate possible overlapping catalytic functionalities or competition with the substrate accessing the active site. However, minimal inhibition by PMSF or E-64 was observed with the purified enzyme (Figure 4C).

### 3.4. Zymography and Protease Purification

Crude and partially purified fractions of the BW26 peptidase were subjected to zymography to visualize proteolytic activity and verify the size of the active protein. Proteolytically active bands were observed in both crude and purified samples, and the activity was inhibited in the presence of 1,10-phenanthroline (Figure 4A), further confirming the presence of a metalloprotease nature of the enzyme. SDS-PAGE of chromatographic fractions showed a prominent band corresponding to the protease in peak 2 (Figure 4B). The proteolytic activity was further characterized using the fluorogenic substrate Mca-Lys-Pro-Leu-Gly-Leu-Dap-(Dnp)-Ala-Arg-NH2. Progress curves indicated that the protease activity was significantly inhibited by 1,10-phenanthroline, while E-64 and PMSF had minimal effects (Figure 4C), consistent with previous findings.

**Figure:**
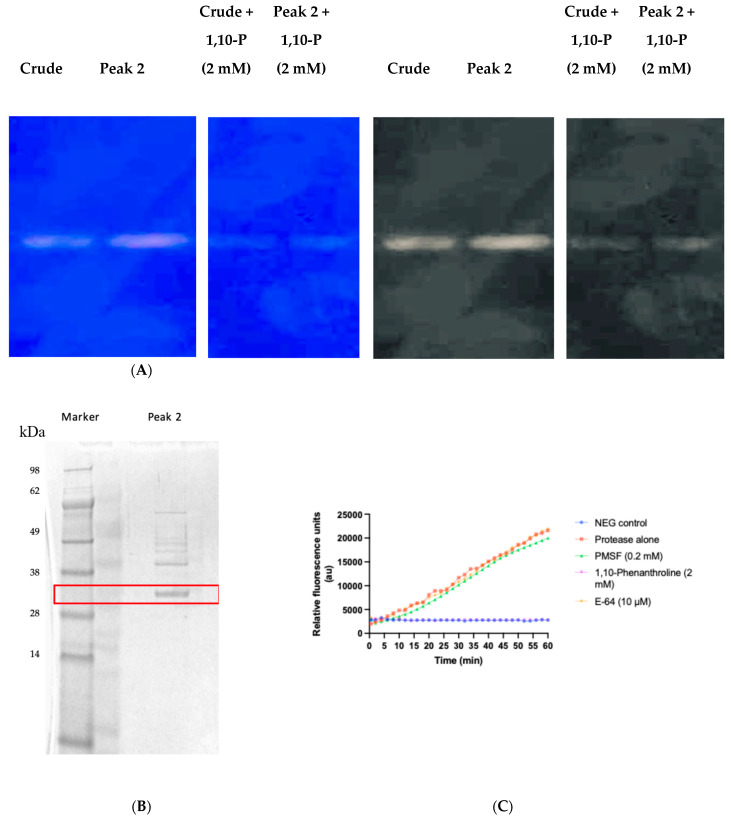


### 3.5. Protease pH and Temperature Profiling

**Figure 5:**
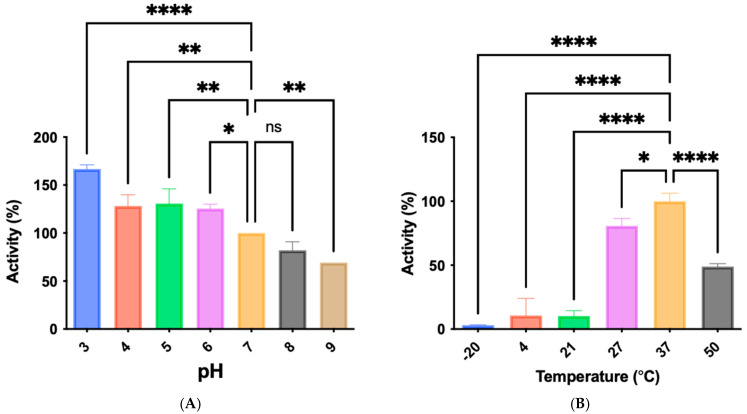
(**A**) pH profiling of azocasein hydrolysis by partially purified fractions of *E. oxidotolerans* protease. (**B**) Temperature-dependent activity of the protease measured by azocasein hydrolysis at different temperatures. Error bars indicate the mean ± standard deviation determined from biological replicates; asterisks indicate significant differences between either pH and 27 °C contol groups, * (*p* < 0.05), **(*p* < 0.01), and **** (*p* < 0.0001) using one-way ANOVA and Dunnett’s post-test analysis (*n* = 3).

The activity of the BW26 protease was assessed across a range of pH values (pH 3 to 9) and temperatures (−20 °C to 50 °C). The enzyme exhibited optimal activity at pH 3, retaining over 80% activity between pH 6.0 and 8.0 (Figure 5A). Temperature profiling revealed optimal activity at 37 °C, with significant activity maintained between 27 °C and 50 °C (Figure 5B).

### 3.6. Protein Sequencing, Structural Analysis, and BLAST Analysis

**Figure 6:**
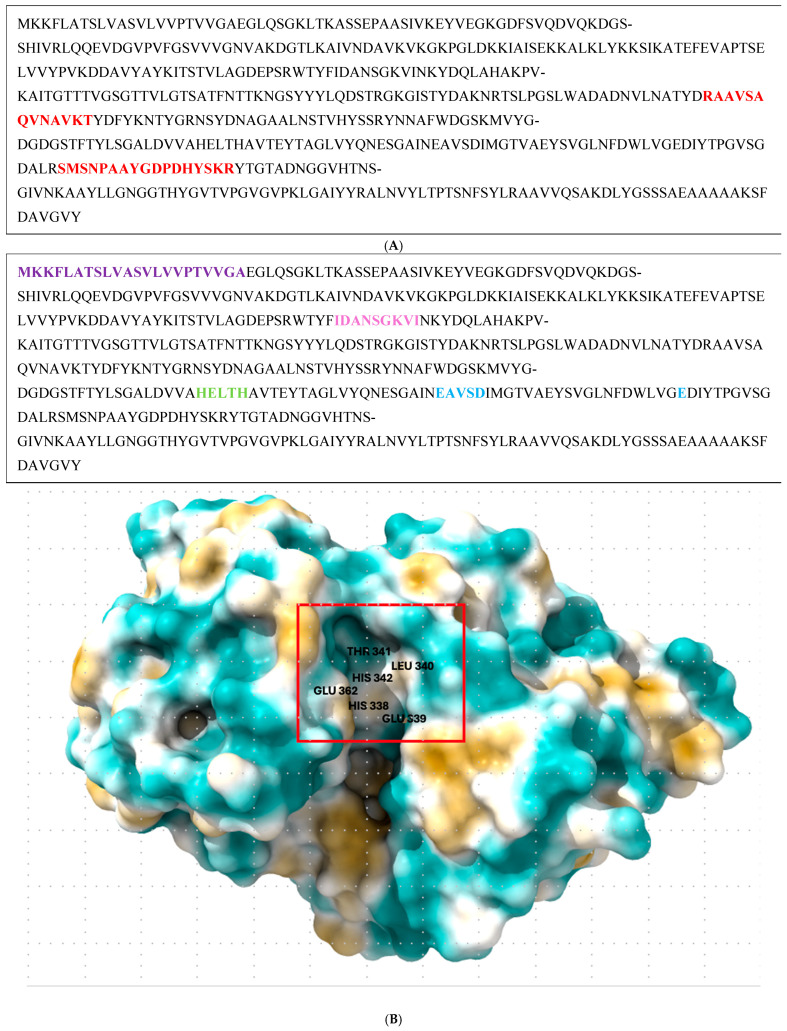

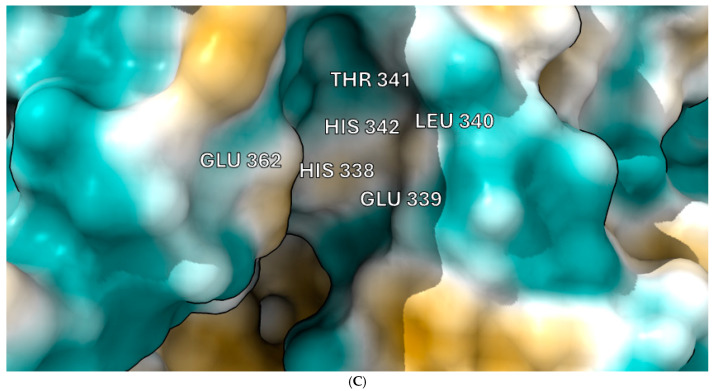
(**A**) Thermolysin protein sequence of *E. oxidotolerans* BW026 starting from amino acid 202; highlighted regions show the peptide fingerprints (13-mer, 17-mer) idenfied by LC-MS-MS. (**B**) MKKFLATSLVASVLVVPTVVGA—predicted signal peptide motif; IDANSGKVI—consistent with the conserved PepSY domain in the pro-peptide of other thermolysin M4 peptidases [45]. HELTH—HEXXH motif in which bound histidine is a zinc ligand and Glu is the active site residue. EAVSD—Glu-Xaa-Xaa-Xaa-Asp motif useful for detecting members of the M4 thermolysin family. (**C**) Active protease structured predicted by AlphaFold v3, showing key residues annotated using ChimeraX.

The protease band excised from SDS-PAGE gels was subjected to LC-MS/MS analysis. The peptides identified matched sequences of thermolysin-like M4-family neutral metalloproteinase (Figure 6A) according to the UniProt database (gene *nprM*, ORF EXIGUO9Y_230090, primary accession A0A6531866). The Mascot score of 141 suggests a very low probability of random match (*p* = 1 × 10^−14.1^), confirming the identity of the protein. The protease sequence was further analyzed using BLAST and UniProt to assess similarity to other known metalloproteases. As outlined in the Supplementary Table, the protease shared a high degree of sequence similarity (85–100%) with M4-family metallopeptidases from various *Exiguobacterium* species, confirming the conservation of thermolysin-like M4 protease across members of the genus. Analysis of the protein sequence revealed conserved motifs characteristic of M4-family metalloproteases, including the HEXXH zinc-binding motif (Jongeneel consensus sequence, first described by Jongeneel et al. [42] and the Glu-Xaa-Xaa-Xaa-Asp motif (Figure 6B). These motifs are essential for catalytic activity and metal ion coordination. The signal peptide was predicted by insertion of the full protein sequence into the SignalP 6.0 server [43,44], and the pro-peptide domain assigned by UniProt. The three-dimensional structure of the protease was predicted using AlphaFold v3 and visualized with ChimeraX (Figure 6C). Key catalytic residues were annotated, and the overall structure was consistent with known thermolysin-like metalloproteases.

BLASTn searches against other *Exiguobacterium* strains showed high sequence identity with thermolysin-like proteases from related species (Supplementary Table S1). *E. oxidotolerans* JCM 12280 showed 100% identity, confirming the conserved nature of the protease within the species.

### 3.7. Inhibitor Screening and Ki Determination

**Figure 7:**
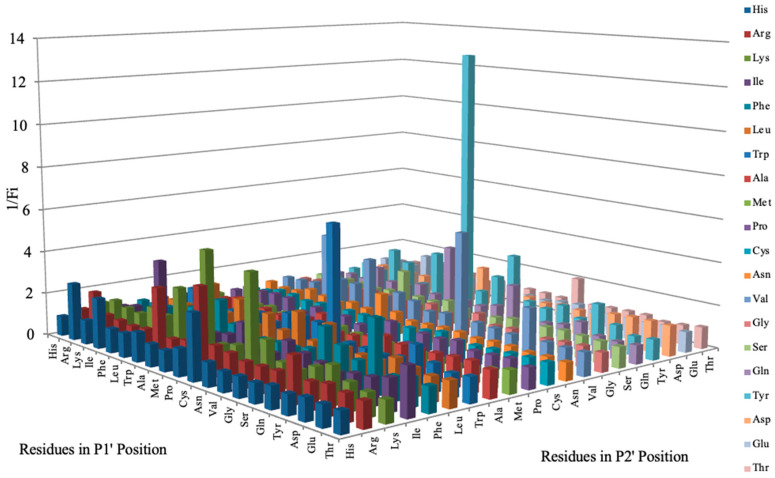
Inhibitor screening results showing 1/Fi values for 400 inhibitors tested against *E. oxidotolerans* thermolysin-like protease. Fi values were derived from substrate hydrolysis progress curves.

To explore potential applications of the protease, a high-throughput inhibitor screening assay was conducted, evaluating a 20 × 20 *N*-alpha dipeptide mercaptoamide inhibitor library against the M4 peptidase from *E. oxidotolerans* (exiguolysin) (Figure 7). From this initial screen, 10 inhibitors (Table 2) were identified as the most potent, and further analysis confirmed them as having the lowest *K*_i_ values (Table 1). The inhibitor SH-CH_2_-CO-Met-Tyr-NH_2_ exhibited the greatest inhibition, with a *K_i_* value of 1.95 μM, indicating a preference for neutral/non-polar, hydrophobic aliphatic amino acids in P1′, although proline, asparagine, and tyrosine feature in the top ten inhibitors in position P1′. The P2′ preference of this enzyme appears to be somewhat broader. In general, the *K*_i_ values reported for the most potent inhibitors from this library against exiguolysin are consistent with those previously reported for both LasB and ZapA [36].

**Table 1:** The *K*_i_ values of the top ten most potent inhibitors of the time-dependent substrate hydrolysis of Mca-Pro-Gly-Leu-Dap(Dnp)Ala-Arg-NH_2_ by the thermolysin-like protease of *E. oxidotolerans*.

Inhibitor	True *K*_i_ (μM)
Me-Met-Tyr-NH_2_	1.95
Me-Met-Lys-NH_2_	3.10
Me-Val-Val-NH_2_	3.21
Me-Pro-Arg-NH_2_	3.97
Me-Asn-Trp-NH_2_	4.35
Me-Tyr-Phe-NH_2_	5.52
Me-Asn-Tyr-NH_2_	5.71
Me-Ile-Val-NH_2_	8.33
Me-Ile-Ile-NH_2_	11.22
Me-Asn-Lys-NH_2_	20.86

### 3.8. Calcium Mobilization in PC-3 and HCT15 Cells

**Figure 8:**
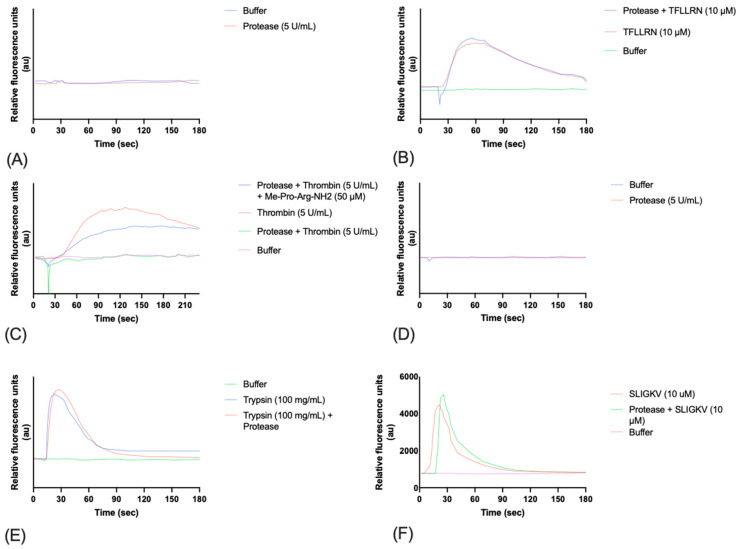
Effect of *E. oxidotolerans* thermolysin-like protease on calcium mobilization in PC-3 cells. (**A**) No calcium mobilization was observed when cells were treated with protease alone. (**B**) PAR-1 activation by TFLLRN (10 μM) was unaffected by protease pre-treatment. (**C**) Protease inhibited thrombin-induced PAR-1 activation, suggesting proteolytic cleavage of the receptor. This disarming effect was reversed by ME-Pro-Arg-NH2. (**D**) No calcium mobilization was observed in HCT15 cells when treated with protease alone. Cells were pre-treated with Fluo-4 Direct calcium dye before addition of the protease. (**E**) Calcium mobilization in HCT15 cells was observed through PAR-2 activation by trypsin (100 ng/mL) following 10 min pre-treatment with thermolysin-like protease, suggesting no inhibitory effect on PAR-2 signalling. (**F**) PAR-2 activation of HCT15 cells by SLIGKV was not impacted by protease treatment, indicating selectivity of the protease for PAR-1 over PAR-2.

The effect of pre-incubation of the protease on calcium mobilization in PC-3 cells (expressing PAR1) was investigated to assess its potential interaction with protease-activated receptors (PARs). Treatment of PC-3 cells with the protease alone did not induce calcium mobilization (Figure 8A). Pre-treatment with the protease did not affect calcium flux induced by the PAR-1 agonist TFLLRN (Figure 8B). However, the protease inhibited thrombin-induced PAR-1 activation, suggesting proteolytic cleavage of the receptor that prevents thrombin binding (Figure 8C). This disarming effect was reversed by pre-incubation of the protease with the inhibitor SH-CH_2_-CO-Pro-Arg-NH2. In HCT15 cells (expressing PAR2), the protease did not impact calcium mobilization induced by the PAR-2 agonist SLIGKV, indicating selectivity towards PAR-1 (Figure 8D–F). These findings highlight the protease’s potential therapeutic application in modulating protease-activated receptor signalling.

## 4. Discussion

This study characterizes a secreted peptidase of *E. oxidotolerans*, with a detailed focus on its biochemical properties, inhibition profile, structural prediction, and potential role in virulence. Through a combination of biochemical assays, inhibitor screenings, structural modelling, and phylogenetic analysis, we provide detailed characterization of this peptidase, tentatively named exiguolysin, as the first representative of a thermolysin-like, M4-family protease from the genus *Exiguobacterium.* Among the strains isolated as part of a wider marine biodiscovery programme, *E. oxidotolerans* BW26 exhibited the greatest caseinolytic activity in a standard culture-based assay (Figure 1A). Initially, casein hydrolysis, observed as clear halos surrounding bacterial colonies on LB agar supplemented with 2% skimmed milk, indicated the secretion of extracellular protease (Figure 1B,C). This is consistent with prior studies showing that casein, a major component of skim milk, is an ideal substrate for identifying protease activity [46]. SDS-PAGE analysis of both crude supernatants of *E. oxidotolerans* growth media and protein extracted from solid growth media following incubation of *E. oxidotolerans* on a nitrocellulose membrane, and partially purified proteins, showed the presence of a dominant protein band at ~32–34 kDa (Figure 1D and Figure 4B). This was subsequently confirmed by casein zymography (Figure 4A).

### 4.1. Classification of Exiguolysin as a Metallopeptidase and Determination of Inhibition Profile

The classification of the exiguolysin as a metalloproteinase was determined by broad spectrum class-specific inhibitors. The protease activity was significantly reduced by 1,10-phenanthroline, in both crude (Figure 3) and partially purified preparations of the peptidase, but unaffected by PMSF or E64, indicating that the catalytic mechanism is that of a metallopeptidase (Figure 4C). This is further corroborated by structural motifs identified from the protein primary structure inferred from the putative gene *nprM* encoding the enzyme (Figure 6B showing the HEXXH zinc-binding motif and Glu-Xaa-Xaa-Xaa-Asp motif), which are characteristic of metallopeptidases and, in particular, M4-family peptidases. However, the weak inhibition by PMSF and E-64 suggests that other catalytic residues, outside of serine or cysteine proteases, might contribute to its activity [47].

The zymographic analysis further confirmed that the proteolytic activity was diminished upon the addition of 1,10-phenanthroline, again supporting the classification of this peptidase as a metalloprotease [48]. Exiguolysin’s activity was retained at high temperatures (50 °C), with optimal activity at 37 °C under acidic conditions, with an optimal pH value of 3 (Figure 5A,B). This is consistent with previous research which has found other acidic, marine-derived proteases to have a similar optimal temperature of activity and pH, and highlighting its adaptability to different environmental conditions [49,50]. This broad temperature and pH range are characteristic of proteases derived from extremophilic or thermophilic organisms and may offer potential biotechnological applications where such broad stability is advantageous. Comprehensive inhibitor profiling of exiguolysin with a 400-member library of *N*-alpha dipeptide mercaptoamides (Figure 7), first described by Cathacart et al. [32,33] and Carson et al. [35,36], identified a number of moderately potent tight binding, reversible inactivators of the enzyme, which may prove to be useful reagents in probing the physiological role of this peptidase in future studies aimed at defining its speculative role in *Exiguobacterium* virulence in vivo. These studies reveal a preference for neutral, non-polar, hydrophobic aliphatic amino acids in P1′ position, with a wider range of amino acids accommodated in the P2′ position.

### 4.2. Protease Purification and Substrate Specificity

The purification of the protease by HIC yielded a highly purified enzyme, as shown by SDS-PAGE analysis, where a single protease band at ~32 kDa was observed (Figure 4B). The enzyme exhibited activity against MMP substrates, particularly Mca-Lys-Pro-Leu-Gly-Leu-Dap-(Dnp)-Ala-Arg-NH2, but no activity was detected with elastase substrates, further supporting its classification as a metalloprotease with substrate specificity dependent on proline residues near the cleavage site [51]. Proline, known for its rigid conformation, may contribute to the enzyme’s specificity and stability [52,53]. Proline’s influence on substrate specificity further supports the enzyme’s classification as a thermolysin-like metalloprotease, which typically displays preferences for substrates with proline residues. *E. oxidotolerans* exhibited moderate beta haemolytic activity on blood agar and against horse erythrocytes. In addition, exiguolysin was able to degrade heat-denatured, but not native, IgG, IgM, and IgA, which could be inhibited by N-alpha dipeptide mercaptoamide inhibitors.

### 4.3. Structural Insights and Phylogenetic Analysis

The structural predictions generated by AlphaFold v3 identified key motifs commonly found in thermolysin-like proteases, such as the HEXXH zinc-binding motif and the Glu-Xaa-Xaa-Xaa-Asp motif (Figure 6A–C). These motifs are critical for the binding of zinc ions and the catalytic activity of metalloproteases [54]. The predicted structural model aligns well with the experimental data, which indicated that metal ion binding is essential for enzymatic activity. The phylogenetic analysis places the protease within the M4 family of thermolysin-like metallopeptidases, according to the MEROPS database [55], with high sequence similarity to other members of the *Exiguobacterium* genus (Figure 2). This is consistent with the identified thermolysin-like proteases in other members of this genus identified in this study bacteria. The high sequence similarity to proteases from other *Exiguobacterium* species supports the classification of the enzyme within this family, though functional differences between these species remain to be explored. Future genomic analysis could provide a more detailed understanding of the evolutionary divergence of these proteases and their specific functional adaptations.

### 4.4. Disarming of PAR-1, but Not PAR2, by Exiguolysin—A Role in Virulence?

The selective modulation of PAR-1 signalling by exiguolysin represents a particularly intriguing finding with potential implications for *E. oxidotolerans* virulence. Given the conservation of thermolysin-like M4 peptidase across the genus (Table 2), and the increasing incidences of clinical cases of infection where *Exiguobacterium* spp. have been implicated, exiguolysin and closely related M4-family peptidases could represent genus-wide virulence factors. Given that PARs play crucial roles in processes such as inflammation and coagulation, the selective disarming of thrombin-induced PAR-1 activation suggests that the protease may be involved in disarming the normal inflammatory response associated with infection. During infection, the production of extracellular proteases by either the host or the pathogen may modify the normal response of protease-activated receptors through cleavage of the extracellular domain or tethered ligands. Consistent with PAR1 disarming/desensitization by exiguolysin in this study, the disarming of PAR1 by thermolysin has been previously demonstrated [56]. The specificity towards PAR-1 (Figure 8A–C), coupled with the lack of effect on PAR-2 signalling (Figure 8D–F), indicates a precise mechanism of action that may represent a specific, highly conserved pathological role for exiguolysin. Thermolysin family (M4) enzymes have a widespread distribution in nature and are implicated in a wide variety of microbial pathogenic processes [31]. Future studies should focus on identification of the cleavage site (presumably within or adjacent to the tether ligand) and demonstration of the activity of exiguolysin in disarming PAR-1 replicated in vivo, to confirm its proposed function as a virulence factor.

## 5. Conclusions

This study provides the first comprehensive characterization of a thermolysin M4-family peptidase secreted by *E. oxidotolerans*: exiguolysin. Through detailed biochemical characterization, analysis of substrate specificity and inhibitor profile, and computational modelling, we propose exiguolysin as a putative virulence factor of *E. oxidotolerans* that is well conserved across the genus. This enzyme may also prove to have applications in biotechnology/biocatalysis given its broad activity range. Exiguolysin demonstrates robust activity in degrading casein, effectively inhibited by 1,10-phenanthroline and *N*-alpha dipeptide mercaptoamide inhibitors, the latter identifying a number of moderately potent reversible inactivators of the enzyme, which may assist in probing the precise physiological role of the enzyme in future studies. The enzyme’s stability across a broad pH and temperature range highlights its adaptability, suggesting potential industrial applications, particularly in environments where such stability is critical. Structural predictions and phylogenetic analysis further support the classification of exiguolysin as a thermolysin M4-family peptidase. Exiguolysin’s selective disarming of PAR-1 signalling is intriguing, indicating a potential role in conditions involving bacterially mediated dysregulated PAR-1 activity, such as blunting of the inflammatory response following infection. This study presents the first comprehensive characterization of exiguolysin, a novel, secreted thermolysin-like M4 peptidase from *E. oxidotolerans*, with evidence of conservation of closely related enzymes across the diverse genus *Exiguobacterium*. Whilst the exact physiological role of exiguolysin is not known, the genus *Exiguobacterium* has been recognized for its potential applications in industry and agriculture, and its members are increasingly implicated as emerging and opportunistic pathogens.

## Figures and Tables

**Table 2:** Structures of the ten most potent inhibitors of the thermolysin-like protease of *E. oxidotolerans*.

(1)	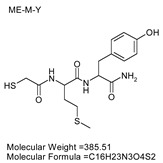	(2)	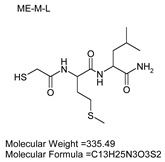
(3)	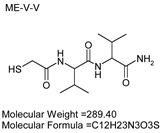	(4)	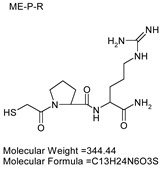
(5)	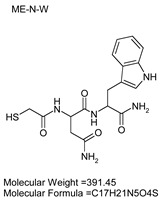	(6)	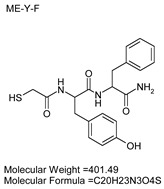
(7)	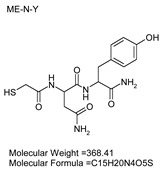	(8)	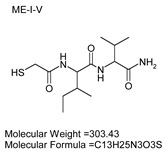
(9)	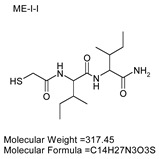	(10)	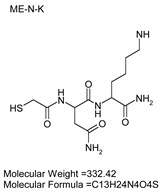

## Data Availability

The original contributions presented in the study are included in the article/Supplementary Material; further inquiries can be directed to the corresponding author.

## Supplementary Materials

The following supporting information can be downloaded at: https://www.mdpi.com/article/10.3390/microorganisms12112311/s1, Table S1: Supplementary Table: BLAST Results of *Exiguobacterium* strains against thermolysin protease to assess similarity and presence.

## Abbreviations

Abz	Aminobenzoic acid
Dap	Diaminopropionic acid
Dnp	2,4-Dinitrophenyl
Fluo	4 AM-Fluorescent Calcium Indicator Acetoxymethyl Ester
HIC	Hydrophobic Interaction Chromatography
Mca	7-methoxycoumarin
MMP	Matrix Metalloproteinase
PAR	Protease-Activated Receptor
PBS	Phosphate-Buffered Saline
PCR	Polymerase Chain Reaction
SDS-PAGE	Sodium Dodecyl Sulphate Polyacrylamide Gel Electrophoresis
TFA	Trifluoroacetic Acid
