# Supplementary material for: Exiguolysin, a Novel Thermolysin (M4) Peptidase from Exiguobacterium oxidotolerans
Source: Microorganisms. 2024 Nov 14;12(11):2311. doi: 10.3390/microorganisms12112311 (PMC11596557; doi:10.3390/microorganisms12112311)
Supplement: Supplementary file 1 [file microorganisms-12-02311-s001.zip › microorganisms-3266191-supplementary.pdf]

Supplementary Table: BLAST Results of *Exiguobacterium* strains against thermolysin protease to assess similarity and presence

Strain ID	Description	Percentage Identity (%)
Exiguobacterium enclense DSM 25128	M4 family metallopeptidase [*Exiguobacterium indicum*]	85.71
Exiguobacterium acetylicum DSM 20416	M4 family metallopeptidase [*Exiguobacterium acetylicum*]	88.65
Exiguobacterium indicum DSM 2840	M4 family metallopeptidase [*Exiguobacterium*]	88.45
Exiguobacterium antarcticum DSM 14480	M4 family metallopeptidase [*Exiguobacterium*]	90.22
Exiguobacterium soli DVS 3Y(T)	M4 family metallopeptidase [*Exiguobacterium*]	90.22
Exiguobacterium oxidotolerans JCM 12280	M4 family metallopeptidase [*Exiguobacterium oxidotolerans*]	100
Exiguobacterium undae DSM 14481	M4 family metallopeptidase [*Exiguobacterium*]	87.48
Exiguobacterium artemiae DSM 16484	No significant similarity found	-
Exiguobacterium sibiricum str. 255-15	M4 family metallopeptidase [*Exiguobacterium artemiae*]	90.61
Exiguobacterium flavidum HF60	M4 family metallopeptidase [*Exiguobacterium flavidum*]	82.07
Exiguobacterium aestuarii DSM:16306	M4 family metallopeptidase *[Exiguobacterium aestuarii*]	73.83
Exiguobacterium qingdaonense S82	M4 family metallopeptidase [*Exiguobacterium qingdaonense*]	70.7
Exiguobacterium marinum DSM 16307	No significant similarity found.	-
Exiguobacterium profundum DSM 17289	M4 family metallopeptidase [*Exiguobacterium*]	73.63
Exiguobacterium algae S126	M4 family metallopeptidase [*Exiguobacterium algae*]	71.29
Exiguobacterium alkaliphilum DSM 21148	No significant similarity found	-
Exiguobacterium aurantiacum DSM 6208	No significant similarity found	-
Exiguobacterium aquaticum JCM 17977	bacillolysin [*Exiguobacterium mexicanum*]	72.8
Exiguobacterium mexicanum DSM 16483	bacillolysin [*Exiguobacterium mexicanum*]	72.8
Exiguobacterium chiriqhucha RW-2	M4 family metallopeptidase [*Exiguobacterium*]	72.41
